# The Iron Distribution and Magnetic Properties of Schistosome Eggshells: Implications for Improved Diagnostics

**DOI:** 10.1371/journal.pntd.0002219

**Published:** 2013-05-16

**Authors:** Stephan Karl, Lucía Gutiérrez, Rafael Lucyk-Maurer, Roland Kerr, Renata R. F. Candido, Shu Q. Toh, Martin Saunders, Jeremy A. Shaw, Alexandra Suvorova, Andreas Hofmann, Michael J. House, Robert C. Woodward, Carlos Graeff-Teixera, Timothy G. St. Pierre, Malcolm K. Jones

**Affiliations:** 1 School of Physics, The University of Western Australia, Crawley, Western Australia, Australia; 2 Laboratório de Biologia Parasitária, Faculdade de Biociências e Laboratório de Parasitologia Molecular, Instituto de Pesquisas Biomédicas, Pontifícia Universidade Católica do Rio Grande do Sul, Porto Alegre, Brazil; 3 School of Veterinary Sciences, The University of Queensland, Australia, and Queensland Institute of Medical Research, Herston, Queensland, Australia; 4 Centre for Microscopy, Characterisation and Analysis, The University of Western Australia, Crawley, Western Australia, Australia; 5 Department of Structural Chemistry, Eskitis Institute for Cell and Molecular Therapies, Griffith University, Brisbane, Australia; George Washington University, United States of America

## Abstract

**Background:**

*Schistosoma mansoni* and *Schistosoma japonicum* are the most frequent causative agents of human intestinal schistosomiasis. Approximately 200 million people in the world are infected with schistosomes. Diagnosis of schistosomiasis is often difficult. High percentages of low level infections are missed in routine fecal smear analysis and current diagnostic methodologies are inadequate to monitor the progress of parasite control, especially in areas with low transmission. Improved diagnostic methods are urgently needed to evaluate the success of elimination programs. Recently, a magnetic fractionation method for isolation of parasite eggs from feces was described, which uses magnetic microspheres to form parasite egg – magnetic microsphere conjugates. This approach enables screening of larger sample volumes and thus increased diagnostic sensitivity. The mechanism of formation of the conjugates remains unexplained and may either be related to specific surface characteristics of eggs and microspheres or to their magnetic properties.

**Methods/Principal Findings:**

Here, we investigated iron localization in parasite eggs, specifically in the eggshells. We determined the magnetic properties of the eggs, studied the motion of eggs and egg-microsphere conjugates in magnetic fields and determined species specific affinity of parasite eggs to magnetic microspheres. Our study shows that iron is predominantly localized in pores in the eggshell. Parasite eggs showed distinct paramagnetic behaviour but they did not move in a magnetic field. Magnetic microspheres spontaneously bound to parasite eggs without the presence of a magnetic field. *S. japonicum* eggs had a significantly higher affinity to bind microspheres than *S. mansoni* eggs.

**Conclusions/Significance:**

Our results suggest that the interaction of magnetic microspheres and parasite eggs is unlikely to be magnetic in origin. Instead, the filamentous surface of the eggshells may be important in facilitating the binding. Modification of microsphere surface properties may therefore be a way to optimize magnetic fractionation of parasite eggs.

## Author Summary

In the present study, we investigated the mechanism underlying a novel diagnostic method for *Schistosoma* – one of the most widespread and frequently occurring parasites infecting humans in tropical countries. In recent years, the world has seen significant reduction in the burden of *Schistosoma* infections in many countries due to improved control and sanitation. However, it is becoming increasingly difficult to evaluate and monitor the progress of control towards elimination. At the moment it is extremely difficult to determine whether the parasite has been eliminated from a region. This is due to the absence of a sensitive and inexpensive method to detect the parasite. A series of recent studies describes a method with vastly improved diagnostic sensitivity based on the magnetic fractionation of parasite eggs from fecal samples. However, the mechanisms of action of this new diagnostic are not currently known. To further optimize and improve this method, we studied the magnetic properties of parasite eggshells and their binding characteristics to magnetic microspheres.

## Introduction

Schistosomiasis is a helminth infection representing a major health burden for humans in tropical and developing nations. Some 200 million people are infected, and 600 million are currently estimated to be at risk of infection [1].

Recently, schistosomiasis control efforts have been increasingly focused on mass drug administration in endemic areas to alleviate morbidity in affected individuals [2]. Although it has been acknowledged that the goal to regularly administer chemotherapy to at least 75% of school-age children at risk of morbidity was not achieved by 2010, many countries are controlling schistosomiasis with increasing success using a combination of therapeutics and improved sanitation [3], [4]. Sustained and effective drug therapy has the effect of pushing the disease into a state of low endemicity, where individuals carry low-level infections that are very difficult to detect. As a result, new efforts are required for parasite surveillance in regions of low endemicity [5].

It has become increasingly recognized that the evaluation and monitoring of control and elimination efforts for schistosomiasis is hindered by the lack of appropriate diagnostic techniques [5], [6]. With a diagnostic limit of approximately 100 eggs per gram feces, the current WHO recommended test, the Kato-Katz method of fecal examination, is limited by poor sensitivity [7], [8], [9]. It is estimated that more than half of all infections with schistosomiasis are missed in cross sectional studies relying on the observation of only one fecal smear, necessitating the need to perform multiple smears [10]. Examining multiple fecal smears at different time points is logistically difficult, and time and labour intensive. There is, therefore, an urgent need to develop new diagnostic methodologies for intestinal schistosomiasis that are highly sensitive and applicable under field conditions [5]. There is also the need for a new gold standard method to which more sensitive, newly developed, simple and field applicable molecular and rapid diagnostic tests can be compared.

Recently, a novel method for *Schistosoma* egg detection based on magnetic fractionation of parasite eggs from fecal matter was developed [11]. For this method, termed Helmintex, magnetic microspheres are added to larger volumes of fecal samples (30 g). Parasite eggs and magnetic microspheres can then be co-purified from other fecal components through the application of a magnetic field and field gradient. The purified egg concentrates are more readily detectable by light microscopy. Teixeira and colleagues reported that magnetic microspheres coated with a variety of adsorbed molecules could be used to purify eggs of *Schistosoma mansoni* from fecal matter in a magnetic field [11]. The nature of the adsorbed molecules had no influence on the efficiency of the purification and even the use of uncoated microspheres resulted in the purification of *Schistosoma mansoni* eggs from feces in a magnetic field. The mechanism of interaction between parasite eggs and microspheres is unclear, yet it is important to characterize it further in order to optimize specificity and efficiency of the Helmintex method [12].

There are two possible explanations for the seemingly specific interaction of magnetic microspheres and *Schistosoma* eggs. Firstly, it could be that biochemical, chemical or physical surface properties of the eggs mediate the interaction. Secondly, the eggs could themselves be magnetic, leading to a magnetically mediated adhesion of the microspheres to the eggs. Since the interaction seems independent of the surface characteristics of the magnetic microspheres, the latter of these two possibilities seemed the more probable at the beginning of this investigation.

It has been shown that the eggshells of *S. japonicum* contain iron in concentrations detectable by energy dispersive X-ray spectroscopy in the transmission electron microscope [13]. The authors of that study suggested that iron assists in the formation of the biopolymer that makes up the eggshells. Eggshells are formed by polymerization of tyrosine-rich eggshell precursor proteins that are synthesised in the vitelline glands of the parasite. The tyrosine residues are oxidised by tyrosinases to *o-*quinones. Lysine and histidine residues on the same or adjacent eggshell precursors subject the *o-*quinones to nucleophilic attack, leading to a robust cross-linked polymer [14], [15], [16].

In other invertebrates, such as the bivalves *Mytilus*, DOPA-rich bonds in quinone-tanned protein polymers are stabilized by divalent metal-ions, including iron [17]. The vitelline glands of schistosomes are enriched with iron and the iron storage protein ferritin [18]. Thus, there is strong evidence for a role of iron in stabilizing the protein polymer that is the schistosome eggshell. Full verification of this hypothesis requires the refinement of methods for hydrolysis of the highly resistant shells [19].

Here we present results of experiments elucidating the elemental composition of the eggshell, with special focus on the organization of iron in the matrix and the resulting magnetic properties of the eggs. We performed a range of experiments to characterise the nature of magnetic microsphere interaction with parasite eggs and in order to investigate whether this interaction was a result of non-specific binding of the microspheres to the surface of the eggs, or whether it was the result of magnetically aided adhesion of eggs and microspheres.

## Materials and Methods

All work using animals was approved by the Animal Ethics Committee of the Queensland Institute of Medical Research (Project P1289). This study was conducted according to guidelines of the National Health and Medical Research Council of Australia, as published in the *Australian Code of Practice for the Care and Use of Animals for Scientific Purposes*, 7th edition, 2004 (www.nhmrc.gov.au). *S. mansoni* was maintained in *Biomphalaria glabrata* snails and *S. japonicum* was sourced from infected *Oncomelania hupensis hupensis* snails collected in Anhui Province, China. Both, *S. mansoni* and *S. japonicum* worm stages were maintained in outbred Swiss mice.

Eggs of both species were purified from livers of mice by digestion of liver parenchymal tissues with collagenase B in phosphate buffered saline (PBS) in the presence of ethylenediaminetetraacetic acid (EDTA) as an iron chelator. The eggs were incubated in enzyme for 8 h at 37°C with agitation. For further purification eggs were centrifuged in Percoll gradients as described by Dalton et al. in 1997 [20].

### Scanning electron microscopy (SEM)

Samples of *S. mansoni* and *S. japonicum* eggs were fixed in 2% (v/v) glutaraldehyde, 1% (w/v) paraformaldehyde in PBS for 60 min at 4°C and washed twice with PBS (pH = 7.4) in 1.5 mL Eppendorf tubes. The samples were then serially dehydrated in ascending concentrations of ethanol (33%, 50%, 66%, and 100% (dry)) followed by two further washes in dry ethanol using a PELCO Biowave microwave processor (TedPella Inc., Redding, CA, USA). Dehydrated samples were transferred onto circular polylysine-coated glass coverslips and critically point dried (Emitech 850 Critical Point Drier, Quorum Technologies, Ashford, UK). The coverslips were then attached to aluminum sample holders and coated with a 5 nm thick platinum coating for morphological analyses. SEM was performed using the in-lens detector of a Zeiss 1555 VP field emission scanning electron microscope operating at 15 keV (Carl Zeiss, Überkochen, Germany).

### Cryopreparation and High Angle Annular Dark Field Scanning Transmission Electron Microscopy (HAADF-STEM)

Eggs of both parasite species were fixed in 3% (v/v) glutaraldehyde in 0.1 M phosphate buffer. Eggs were transferred to a solution of 20% (w/v) bovine serum albumin in PBS on a copper membrane and rapidly frozen in a Leica EM PACT2 High Pressure Freezer (Leica, Vienna, Austria). Subsequently, the membranes and samples were transferred in cryo-tubes under liquid nitrogen to a Leica EM AFS freeze substitution apparatus for fixation and dehydration in 2% (w/v) osmium tetroxide and 0.5% uranyl acetate (w/v) in 100% anhydrous acetone. The tissues were cryo-substituted for 3 days, according to the following protocol: The temperature of the substitution chamber was increased from −160°C to −85°C over 2 h, and maintained at −85°C for 48 h, after which the samples were brought to room temperature. After further washes in anhydrous acetone, the samples were infiltrated and embedded in Epon resin.

For HAADF-STEM, resin sections were cut from blocks at a thickness of 150 nm using an EM UC6 ultramicrotome (Leica, Vienna, Austria) and mounted onto 200 µm mesh carbon filmed copper grids for analysis at 300 kV using a JEOL JEM 3000F FEGTEM transmission electron microscope (JEOL, Tokyo, Japan). A ∼1 nm probe size was used to image the mass variation within the sections, with areas of high mass appearing bright. Energy Dispersive X-ray Spectroscopy (EDS) data was combined with STEM imaging using an Oxford Instruments INCA detector (Oxford Instruments NanoAnalysis, High Wycombe, UK) to map the composition of features of interest.

### Superconducting quantum interference device (SQuID) magnetic susceptometry

SquiD magnetic susceptometry is a technique to determine the magnetic properties of any given solid material. Samples are exposed to a desired sequence of magnetic fields at constant temperature or a sequence of temperatures at a constant magnetic field. The magnetization of the sample material resulting from this exposure is recorded for each point in a sequence. Using standard sequences, basic magnetic properties (e.g., whether a material is ferromagnetic, paramagnetic or diamagnetic (non-magnetic)) can be determined.

Lyophilized *S. mansoni* and *S. japonicum* eggs were placed in gel capsules for magnetic characterization in a 7 Tesla (T) magnetic property measurement system SQuID magnetic susceptometer (Quantum Design, San Diego, CA, USA). Magnetic hysteresis loops were acquired between −7 T and 7 T at 5 K. Zero-field-cooled and field-cooled (ZFC-FC) magnetization versus temperature curves were obtained from 5 to 300 K, in a measurement field of 0.01 T.

### Inductively coupled plasma atomic emission spectroscopy (ICP-AES)

The concentration of iron, copper and silicon was determined for both types of eggs using ICP-AES. Inductively coupled plasma atomic emission spectroscopy (ICP-AES) is an analytical technique used to determine the elemental composition of a material. It uses inductively coupled plasma to produce excited atoms and ions that emit electromagnetic radiation at wavelengths characteristic of a particular element which are then detected by a detector.

The same samples as in the SQuID measurements were used. For ICP-AES analysis, three replicate samples were digested in 70% HNO_3_ at 95°C. The analysis was performed at the Marine and Freshwater Research Laboratory at Murdoch University, Murdoch, WA, Australia.

### Exposure of eggs to magnetic fields with and without magnetic microspheres

In order to assess the ability to manipulate parasite eggs using a magnetic field, approximately 100 glutaraldehyde-fixed eggs of *S. japonicum* were floated on a 100% Percoll/water interface in a 5 mL cell culture dish. No spontaneous hatching of eggs was observed in these fixed eggs. A cylindrical neodymium-iron-boron magnet was brought close to the eggs so that they were exposed to a magnetic field of approximately 0.1 T and a magnetic field gradient of approximately 35 T/m while being observed under an optical microscope.

To assess the capability of the different egg species to bind magnetic microspheres, eggs of the two species were incubated with microspheres at egg/microsphere ratios of 1∶100 and 1∶500. Unbound microspheres were washed out using custom made filters after an incubation time of 10 min with agitation. Images of the conjugated microsphere/egg suspension were taken at a 100-fold magnification and the distributions of the number of observed microspheres per egg were recorded and compared with the Poisson distribution.

### Analysis of magnetic susceptometry data

The SQuID magnetometry data was fitted to two functions, the Brillouin function and Curie's law. These functions are used to determine the atomic iron specific moment in the samples [21]. The Brillouin function is specifically used to describe the response of an ideal paramagnet to an applied magnetic field. The spin state and thus the oxidation state of the iron atoms in a material can be deduced from the Brillouin fit using Equation 1.
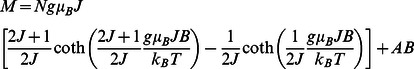
(1)The function describes the dependency of the magnetization (*M*) on the applied magnetic field (*B*) in an ideal paramagnet mixed with diamagnetic (non-magnetic) atoms and gives the total angular momentum quantum number *J* of the microscopic paramagnetic moments of the material. *N* is the number of paramagnetic atoms in the sample; *g* is the electron *g*-factor (−2.0023); *μ_B_* is the Bohr Magneton (9.274×10^−24^ J T^−1^); *J* is the total angular momentum quantum number for each paramagnetic atom; *k_B_* is the Boltzmann constant (1.380×10^−23^ m^2^ kg s^−2^ K^−1^); *T* is the temperature and *B* is the magnetic flux density. The factor *A* is the diamagnetic susceptibility of the sample holder and the diamagnetic components of the eggs.

Curie's law is used to describe the temperature dependency of magnetic susceptibility (*χ*). Data for this analysis are often plotted as *1/χ* versus *T*. A linear relationship with line of best fit running through the coordinate origin indicates ideal paramagnetic behavior. In the present study, the magnetization versus temperature data were fitted with Curie's law shown in Equation 2.
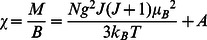
(2)where *χ* is the magnetic susceptibility of the eggs and all other symbols correspond to those used in Equation 1.

## Results

### Scanning electron microscopy

**Figure 1:**
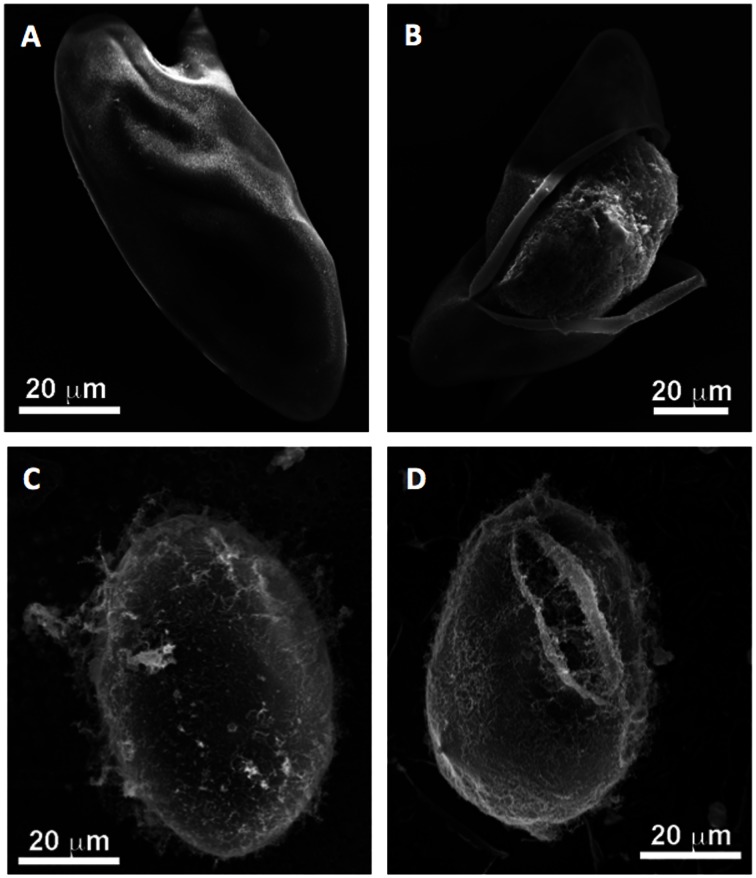
Morphology of *Schistosoma mansoni* and *Schistosoma japonicum* eggs. Panel A shows an intact egg of *S. mansoni*. Panel B shows an *S. mansoni* egg broken open with the miracidium still inside the egg. Panels C and D show similar images for *S. japonicum*.

SEM images of *S. mansoni* and *S. japonicum* eggs are shown in Figure 1. The eggs exhibit the typical features of *S. mansoni* (large spine) and *S. japonicum* (oval shape, small spine) [22]. The fibrous matrix surrounding the egg, also typical for *S. japonicum*, can clearly be seen in Figures 1C and 1D [22]. Figures 1B and 1D show examples of eggs of both species where the eggshell has broken open and the miracidium is still inside the egg. It can be seen that the eggshell curls outwards after initial rupture.

**Figure 2:**
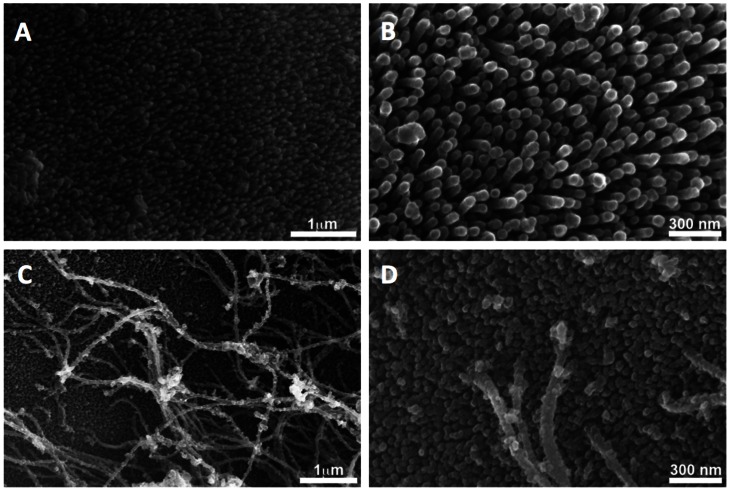
Surface characteristics of *Schistosoma mansoni* and *S.* eggshells. Panels A and B show the surface ofjaponicum a S. *mansoni* egg imaged with high resolution scanning electron microscopy illustrating that the surface is completely covered with filaments or microspines. Figures C and D show similar observations of *S. japonicum*. The microspines on the surface of *S. japonicum* are shorter and the surface is covered with an additional filamentous matrix.

Figure 2 shows details of the surfaces of the eggs of both species at a higher magnification. The surface of *S. mansoni* is covered with evenly spaced structures previously termed microspines with a length of about 200–300 nm and a diameter of 60 nm [22], [23]. The surface of *S. japonicum* is also covered with microspines, however they are considerably shorter. In addition a structure, previously termed the fibrous matrix covers the *S. japonicum* surface [22].

### Transmission electron microscopy

**Figure 3:**
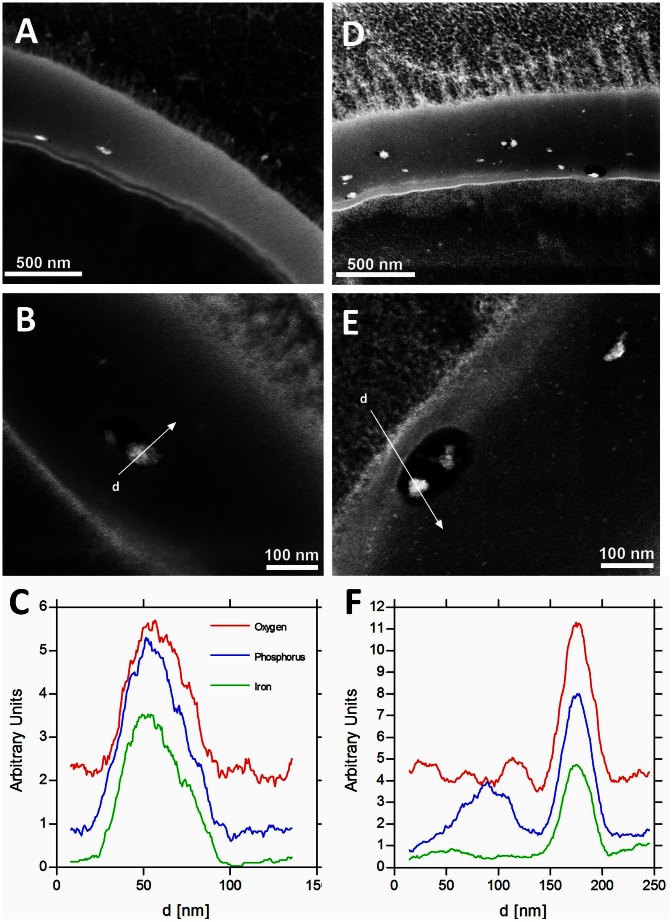
Iron localization within the *Schistosoma* eggshell. Panel A shows inclusions of iron phosphate in the shell of *S. mansoni* at low resolution. Panel B shows similar inclusions in *S. mansoni* at a higher resolution. Panel C depicts the STEM-EDS spectra for iron, phosphorous and oxygen acquired when scanning across an inclusion, along the white line (d) shown in Panel B. Panels D, E and F show similar observations for *S. japonicum*.

The elemental composition and structure of the eggshells of both species was studied by HAADF-STEM and STEM-EDS (Figure 3). The TEM images show that the eggshells are about 700 nm thick. There are regions where the shell is interspersed with small holes of variable size (50–200 nm). These holes have been shown to be empty in other studies [23]. In the present study we show for the first time that these holes are partially filled with a material containing iron, phosphorous and oxygen and we hypothesise that this material is an iron-phosphate that is retained more readily by cryo-fixation and subsequent freeze-substitution processing compared to conventional TEM sample preparation methods (Figure 3).

### Magnetic measurements

**Figure 4:**
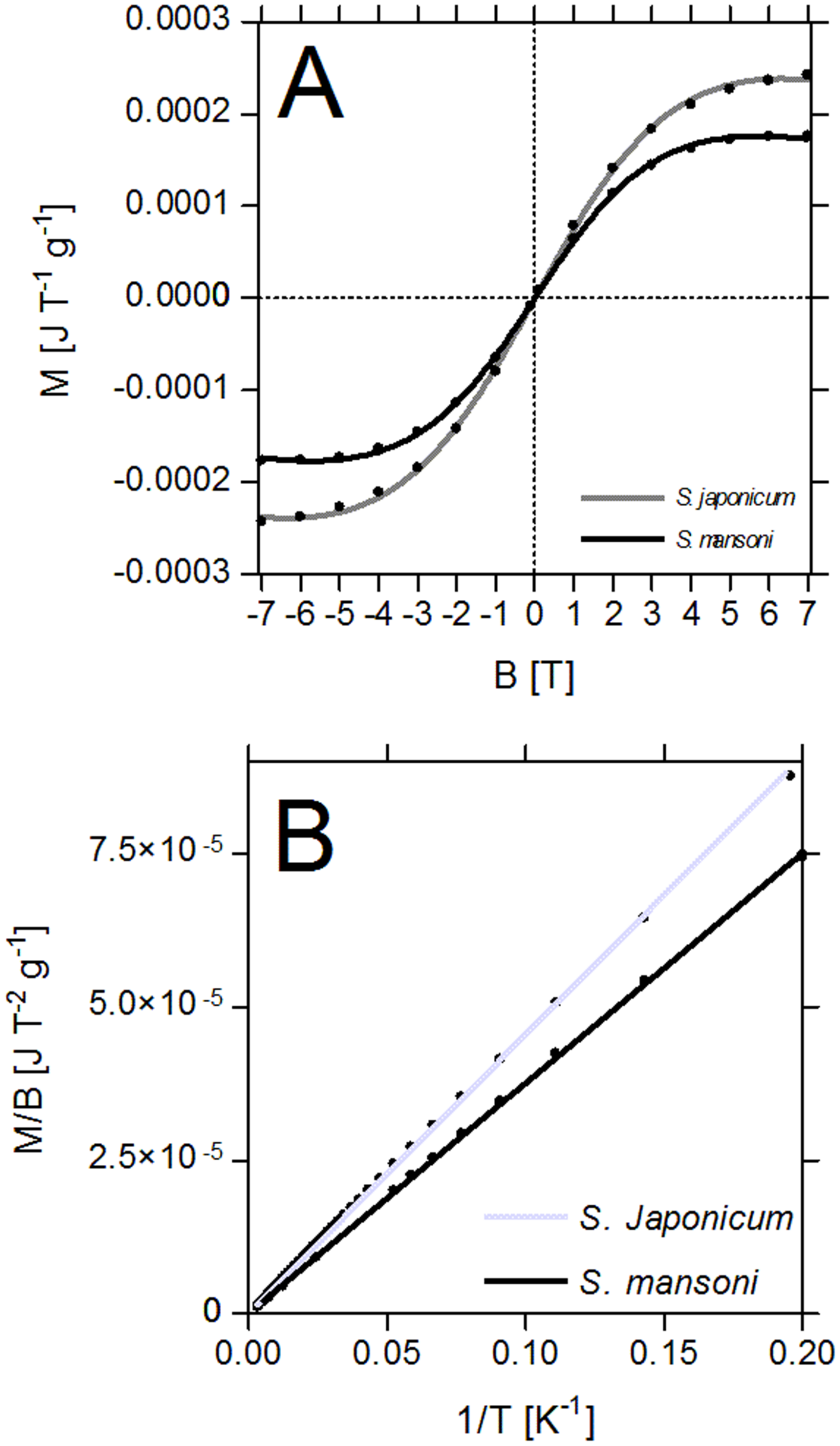
Results of magnetic susceptibility analysis. Figure 4A shows the curve fits of the Brillouin function with diamagnetic contribution (Equation 1) to the magnetization versus magnetic field data (for whole eggs at a temperature of 5 K). It can be seen that ideal paramagnetic behavior is approximated with good precision. (R^2^>0.99) for both, *Schistosoma mansoni* and *Schistosoma japonicum* eggs. Figure 4B shows the curve fits of Curie's law to the magnetization versus inverse temperature data. Again, ideal paramagnetic behavior can be observed. For *S. mansoni* the total magnetic moments were approximated to 4.84×μ_B_ using the Brillouin function and 4.76×μ_B_ using Curie's law. For *S. japonicum* the total magnetic moments were approximated to 4.12×μ_B_ using the Brillouin function and 3.71×μ_B_ using Curie's law.

The results from the magnetometry measurements of the eggs are presented in Figure 4. Both, *S. mansoni* and *S. japonicum* eggs exhibited paramagnetic behaviour with no hysteresis at 5K (Figure 4A). The magnetic susceptibility versus temperature measurements were in nearly perfect agreement with Curie's Law (Figure 4B). The magnetic moment per iron atom (measured in Bohr magnetons - μ_B_) obtained from fitting the Brillouin function with an additional diamagnetic contribution (Equation 1) to the magnetization versus magnetic field data (Figure 4A) at 5K was 4.8 and 4.1×μ_B_ for *S. mansoni* and *S. japonicum* respectively. It was assumed that iron was the only paramagnetic material in the eggs. Similar values were obtained from fits of Curies's law to the magnetization versus temperature data (4.76 and 3.7×μ_B_ for the *S. mansoni* and *S. japonicum* eggs respectively). These values agree but are slightly lower than what would be expected if all the iron in the sample were present as high spin Fe^2+^ ions (typically 5.4×μ_B_) or high spin Fe^3+^ (typically 5.9×μ_B_). We can therefore conclude that there is mix of high spin and low spin iron configurations present in the eggs. Further, more sophisticated measurements using, for example, Mössbauer spectroscopy, that can be used to detect the chemical environment around each iron atom, would be necessary to resolve the exact distribution of these configurations.

### Elemental analysis using inductively coupled plasma atomic emission spectroscopy (ICP-AES)

ICP-AES data are summarized in Table 1. *S. mansoni* and *S. japonicum* eggs contained approximately 0.74 mg/g and 1.26 mg/g of iron respectively (dry weight). By comparison, 1 g of blood contains 3.39 mg iron and 1 g of normal human feces approximately 0.3 mg iron (dry weight) [24]. The concentrations of copper and silicon were also determined.

**Table 1:** Elemental concentration in *Schistosoma mansoni* and *Schistosoma japonicum* eggs.

	Fe (mg/g)	Si (mg/g)	Cu (mg/g)
*S. mansoni*	0.74	0.15	0.11
*S. japonicum*	1.26	0.03	0.03

### Exposure of eggs to magnetic fields with and without microspheres

**Figure 5:**
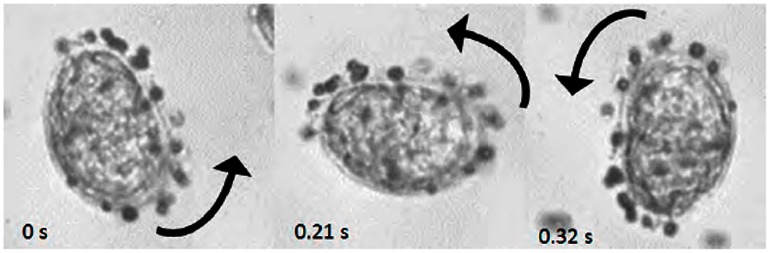
*Schistosoma mansoni* egg – paramagnetic microsphere conjugates. At least 15 microspheres can be seen bound to the surface of the egg. A magnet is rotated around the suspension by 180 degrees over approximately 0.5 seconds (black arrows indicate the movement of the magnet). The images represent frame captures from Video S1 available as supporting information.

**Figure 6:**
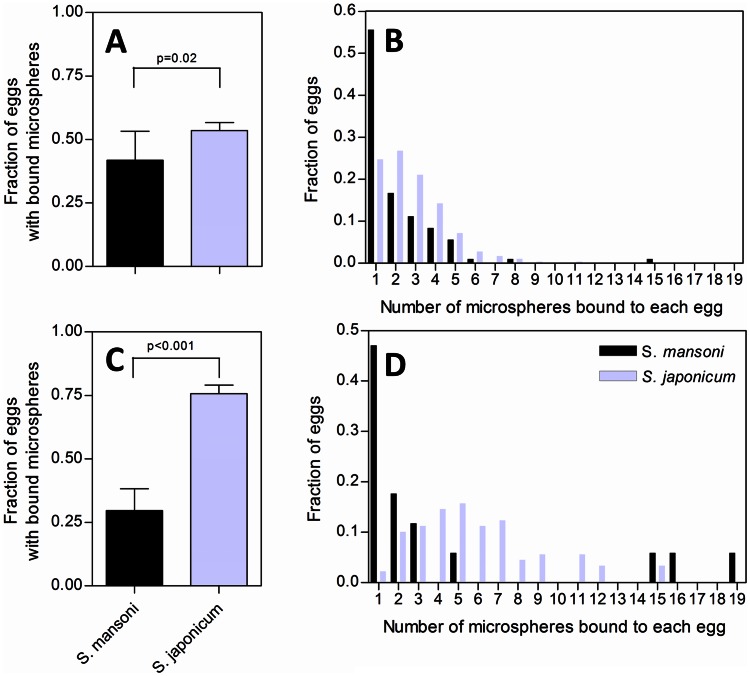
Microsphere binding characteristics to *S.* mansoni and *S. japonicum* eggs. Panel A shows the fraction of eggs that had at least one microsphere bound at an egg to microsphere ratio of 1∶100. Panel B shows the distribution of the number of microspheres bound to eggs of the two parasite species at an egg to microsphere ratio of 1∶100. Panels C and D show the same data for an egg to parasite ratio of 1∶500. For both ratios *S. japonicum* eggs spontaneously conjugated with microspheres at a significantly higher frequency than *S. mansoni* eggs. Similarly, the average number of microspheres per individual egg was considerably higher for *S. japonicum* than for *S. mansoni* (Panels B and D).

No movement of parasite eggs suspended at the Percoll/water interface was observed when the eggs were exposed to a strong magnetic field and a high magnetic field gradient, and imaged with light microscopy. However, when magnetic microspheres were incubated with parasite eggs they readily bound to a fraction of the eggs even without the presence of a magnetic field. The microsphere-egg conjugates were very susceptible to magnetic fields and field gradients as shown in Figure 5 and the video file provided as supporting information (Video S1). Figure 6 illustrates the binding characteristics of the microspheres to the parasite eggs. For both tested parasite egg to microsphere ratios (1∶100 and 1∶500) the fraction of *S. japonicum* eggs that bound microspheres was statistically significantly higher than the fraction of *S. mansoni* eggs that bound microspheres (54% versus 41%, p = 0.02 for the 1∶100 ratio and 76% versus 30%, p<0.001, for the 1∶500 ratio, unpaired t-test). In addition the number of microspheres which bound to individual *S. japonicum* eggs was significantly higher than that for *S. mansoni* (Figure 6). The distribution of microspheres per egg was not well characterized by a single Poisson distribution, especially for the *S. japonicum* eggs but reasonable fits were obtained on the assumption that a fraction of the eggs had no binding capacity (for more details on the analyses using Poisson statistics please refer to the supporting Figures S1 and S2 as well as Text S1).

## Discussion

The present study investigated the magnetic properties as well as iron localization and content of *S. mansoni* and *S. japonicum* eggs and especially the eggshells. This investigation was conducted to elucidate the processes underlying the success of a previously developed magnetic fractionation approach for the detection of parasite eggs in fecal samples, namely the Helmintex method [11]. In recent years, this method has been used in a series of diagnostic studies and has consistently shown improved sensitivity when compared with the conventional Kato-Katz method of fecal evaluation and the saline gradient method [11], [25], [26], [27].

The most important question for optimizing the existing Helmintex method was whether the magnetic properties of the *Schistosoma* eggs were the cause for the adhesion of the magnetic microspheres to the surface of the eggs or whether this binding was of another biochemical or physical nature.

We show that the eggshells of *S. mansoni* and *S. japonicum* eggs contain iron in concentrations measurable by STEM-EDS and ICP-AES, and that the eggs are distinctly paramagnetic, meaning they magnetize in an applied magnetic field and demagnetize when the magnetic field is taken away. These are the principal characteristics required for magnetic fractionation. Interestingly, most of the iron seems to be accumulated as iron-phosphate in pores in the eggshell.

However, the magnetization of the eggs was comparatively weak and the iron content was low. Furthermore we did not observe any movement of the eggs in magnetic fields and field gradients of a similar order of magnitude to those used in the Helmintex protocol. Therefore, the interaction between the eggs and the magnetic microspheres, which is the basis of the success of the Helmintex method, is unlikely to be magnetic in origin.

The original Helmintex studies have shown that magnetic microspheres coated with a wide variety of different ligands could be used to purify parasite eggs [11]. Here, we show for the first time that microspheres physically bind to the eggs. The high surface area of the filamentous outer structure of the eggs may be part of the explanation as this large surface area may provide strong overall adhesion from relatively weak interactions. This hypothesis is further supported by the observation that *S. japonicum* eggs with their additional fibrous matrix bound significantly more microspheres than *S. mansoni* eggs, which do not have this matrix. However, the distributions of microspheres per egg observed in the binding studies suggest that a fraction of the eggs have very little, if any, binding capacity. Furthermore, it should be noted that the fixation using glutaraldehyde may lead to modified surface characteristics of the eggs. Further studies are required to investigate the impact of fixation on particle binding.

The present study provides the first magnetic characterization of *S. mansoni* and *S. japonicum* eggs. We report the discovery of an iron-containing material, presumably iron phosphate located in pores within the eggshell. We provide evidence that *Schistosoma* eggs are not magnetic enough to move in an applied magnetic field of a similar order of magnitude as the one used in the Helmintex method. We show that magnetic microspheres spontaneously bind to eggs of *S. mansoni* and, to a greater degree, to *S. japonicum*. Based on these results we conclude that the conjugation of magnetic microspheres and parasite eggs is mediated not by magnetism but by the surface properties of eggs and microspheres. Systematic quantification of the binding of microspheres that have different surface functionalizations to parasite eggs is likely to represent an opportunity to optimize the Helmintex magnetic fractionation method. Previous field studies have indicated that such an optimized Helmintex method may be developed into a new gold standard to validate future rapid diagnostic and molecular methods for *Schistosoma* detection [25], [26], [27].

## Supporting Information

Figure S1 **Comparison of the calculated Poisson distribution (red) and measured distribution of microspheres per egg (black) at a microsphere to egg ratio of 100 microspheres per egg.** Panel A shows the distribution of the number of microspheres bound to all *S. mansoni* eggs (including those eggs that had no microspheres bound). Panel B shows the distribution for *S. mansoni* when the eggs that had no microspheres bound to them were excluded. Panel C shows the distribution of the number of microspheres bound to all *S. japonicum* eggs (including those eggs that had no microspheres bound). Panel D shows the distribution for *S. japonicum* when the eggs that had no microspheres bound to them were excluded.
(TIF)

Figure S2 **Comparison of the calculated Poisson distribution (red) and measured distribution of microspheres per egg (black) at a microsphere to egg ratio of 500 microspheres per egg.** Panel A shows the distribution of the number of microspheres bound to all *S. mansoni* eggs (including those eggs that had no microspheres bound). Panel B shows the distribution for *S. mansoni* when the eggs that had no microspheres bound to them were excluded. Panel C shows the distribution of the number of microspheres bound to all *S. japonicum* eggs (including those eggs that had no microspheres bound). Panel D shows the distribution for *S. japonicum* when the eggs that had no microspheres bound to them were excluded.
(TIF)

Video S1 **Movement of microsphere-egg conjugates when exposed to a magnetic field of approximately 0.1 T and a field gradient of approximately 35 T/m.**
(WMV)

Text S1 **Additional information on the statistical analysis of binding of magnetic microspheres to** ***S. mansoni*** **and** ***S. japonicum*** **eggs providing further explanations for figures S1 and S2.**
(DOC)
