# Supplementary figures and images for: The Iron Distribution and Magnetic Properties of Schistosome Eggshells: Implications for Improved Diagnostics
Source: PLoS Negl Trop Dis. 2013 May 16;7(5):e2219. doi: 10.1371/journal.pntd.0002219 (PMC3656142; doi:10.1371/journal.pntd.0002219)

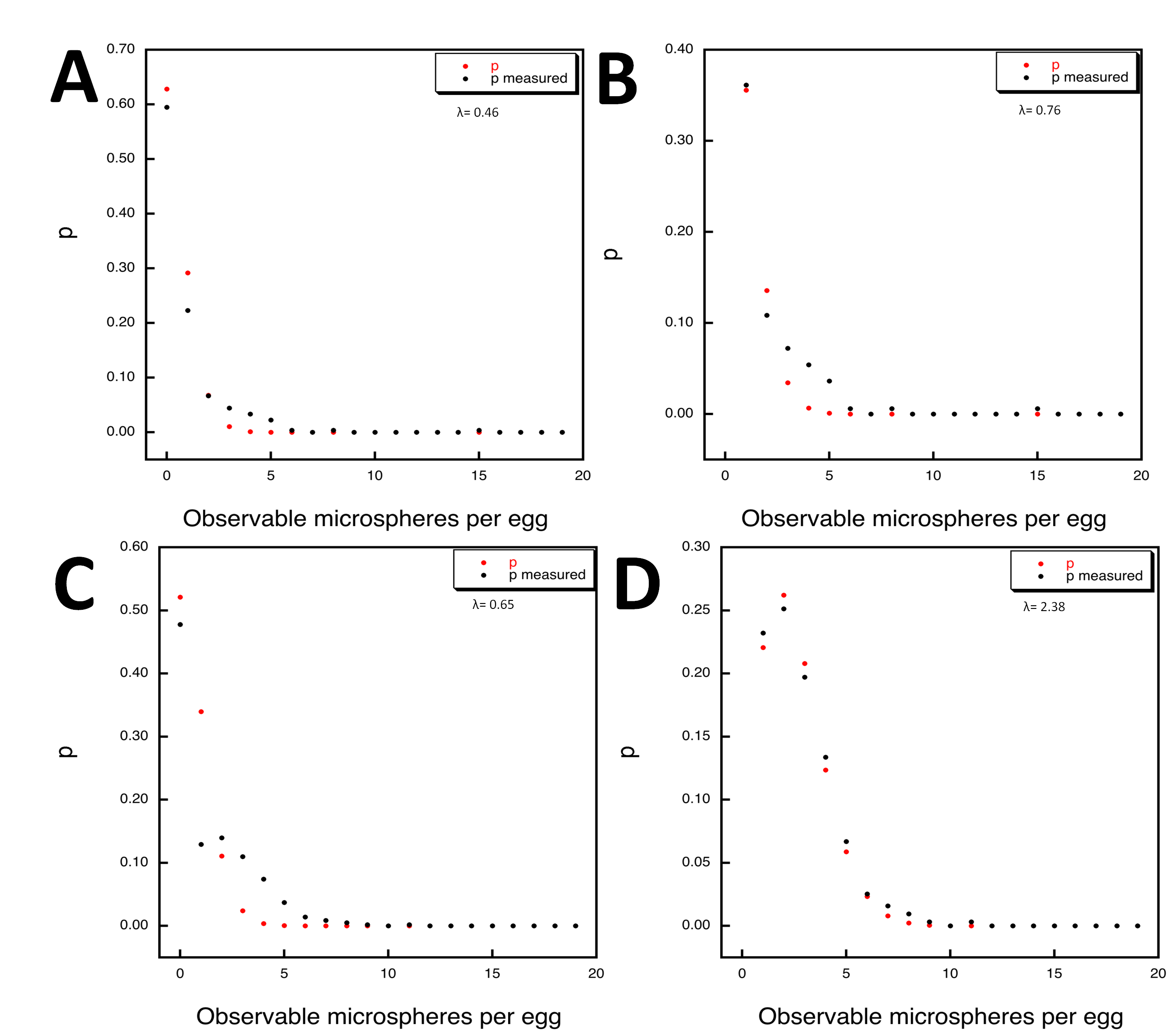

Supplement: Figure S1 — Comparison of the calculated Poisson distribution (red) and measured distribution of microspheres per egg (black) at a microsphere to egg ratio of 100 microspheres per egg. Panel A shows the distribution of the number of microspheres bound to all S. mansoni eggs (including those eggs that had no microspheres bound). Panel B shows the distribution for S. mansoni when the eggs that had no microspheres bound to them were excluded. Panel C shows the distribution of the number of microspheres bound to all S. japonicum eggs (including those eggs that had no microspheres bound). Panel D shows the distribution for S. japonicum when the eggs that had no microspheres bound to them were excluded. (TIF) [file pntd.0002219.s001.tif]

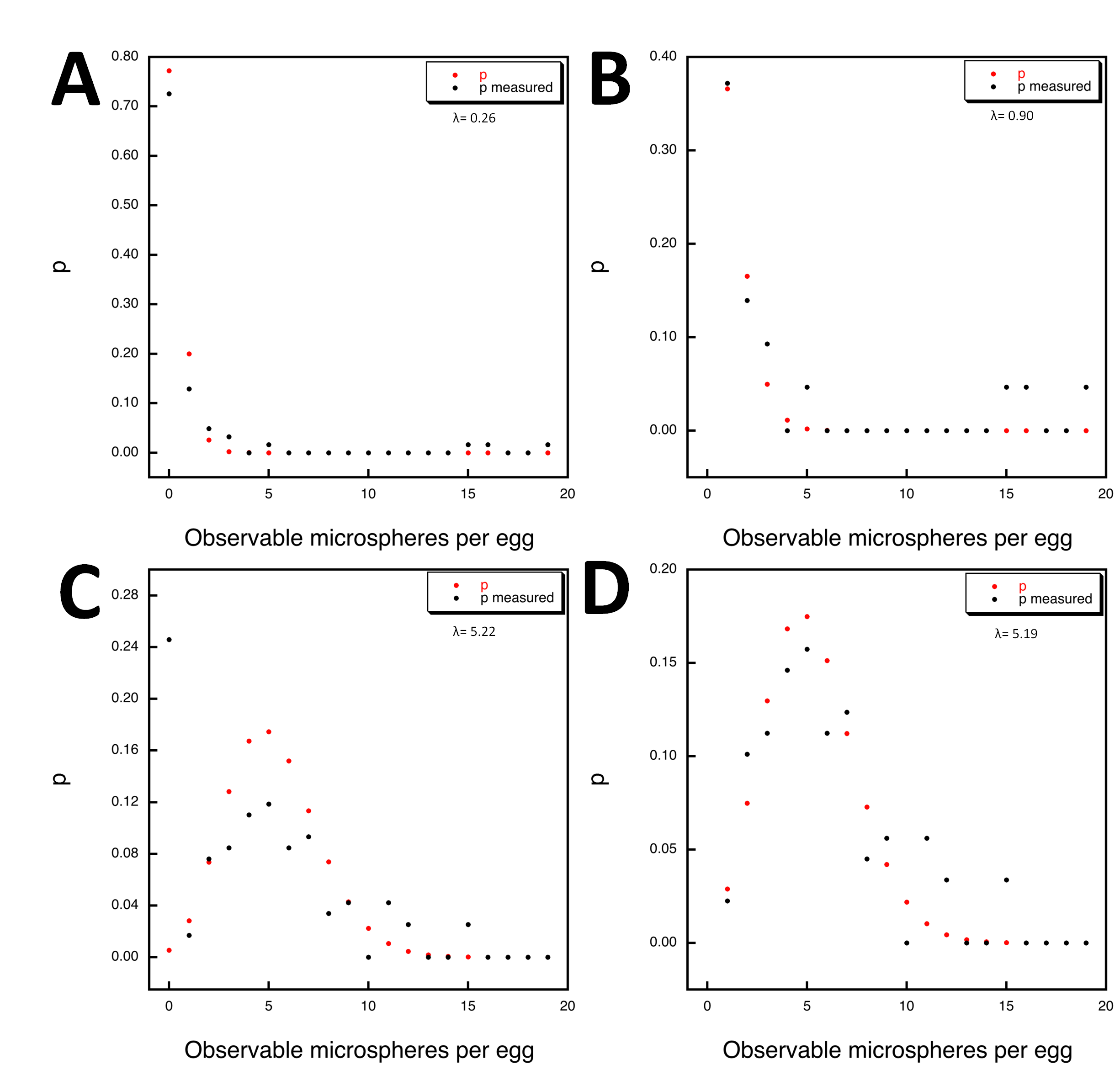

Supplement: Figure S2 — Comparison of the calculated Poisson distribution (red) and measured distribution of microspheres per egg (black) at a microsphere to egg ratio of 500 microspheres per egg. Panel A shows the distribution of the number of microspheres bound to all S. mansoni eggs (including those eggs that had no microspheres bound). Panel B shows the distribution for S. mansoni when the eggs that had no microspheres bound to them were excluded. Panel C shows the distribution of the number of microspheres bound to all S. japonicum eggs (including those eggs that had no microspheres bound). Panel D shows the distribution for S. japonicum when the eggs that had no microspheres bound to them were excluded. (TIF) [file pntd.0002219.s002.tif]
