# Supplementary material for: The Iron Distribution and Magnetic Properties of Schistosome Eggshells: Implications for Improved Diagnostics
Source: PLoS Negl Trop Dis. 2013 May 16;7(5):e2219. doi: 10.1371/journal.pntd.0002219 (PMC3656142; doi:10.1371/journal.pntd.0002219)
Supplement: Text S1 — Additional information on the statistical analysis of binding of magnetic microspheres to S. mansoni and S. japonicum eggs providing further explanations for figures S1 and S2. (DOC) [file pntd.0002219.s004.doc]

**Supplementary information on the statistical analysis of binding of magnetic microspheres to *S. mansoni* and *S. japonicum* eggs**

Figures S1 and S2 show fits of the Poisson distribution to the distributions of microspheres per egg in the experiments where microspheres were mixed with eggs in ratios of 100 and 500 microspheres per egg. A Poisson distribution of observed numbers of microspheres per egg could be expected if each egg in a population has the same affinity for microspheres. In Figures S1 and S2, *P* is the probability of observing the given number of microspheres on a randomly selected egg and *λ* is the number of microspheres bound to an egg expected by the model and is related to the affinity of the eggs for microspheres.

The reason for carrying out this analysis was to determine whether the observation that some eggs have no microspheres bound is either *(a)* because of random fluctuations in the number of eggs binding (i.e. Poisson statistics) or *(b)* because not all eggs have the same affinity for microspheres (i.e. some may have no affinity at all). The Poisson model generally predicts a finite probability of observing eggs with no microspheres even if all eggs have an affinity for microspheres because there will always be a random chance that a given egg does not meet a microsphere. Each data set has been analysed twice. In the first analysis, all eggs are considered. In the second analysis, the Poisson distribution is fitted only to the data for eggs with observable bound microspheres. In the case of *S. japonicum*, in particular, the fits to the data for all eggs are very poor while fits only to the bound egg data are reasonable. For example, when considering the data point for zero microspheres per egg for the *S. japonicum* eggs in the 500 ratio experiment (Figure S2 C), the predicted number of eggs with zero observable microspheres is significantly less than the number observed. This suggests that there are two populations of eggs within the sample, those that have a characteristic binding affinity for the microspheres and those that do not. From this analysis it can be concluded that, especially in the case of *S. japonicum*, it appears that there are two populations of eggs, those that have an affinity for microspheres and those that do not. Further studies are required to elucidate what distinguishes these two populations.
